# GLP-1 receptor agonists (GLP-1RAs): cardiovascular actions and therapeutic potential

**DOI:** 10.7150/ijbs.59965

**Published:** 2021-05-11

**Authors:** Xiaoxuan Ma, Zhenghong Liu, Iqra Ilyas, Peter J. Little, Danielle Kamato, Amirhossein Sahebka, Zhengfang Chen, Sihui Luo, Xueying Zheng, Jianping Weng, Suowen Xu

**Affiliations:** 1 Institute of Endocrine and Metabolic Diseases, The First Affiliated Hospital USTC, Division of Life Sciences and Medicine, University of Science and Technology of China, Hefei, 230001, China.; 2 Sunshine Coast Health Institute, University of the Sunshine Coast, Birtinya, QLD 4575, Australia.; 3 School of Pharmacy, Pharmacy Australia Centre of Excellence, the University of Queensland, Woolloongabba, Queensland 4102, Australia.; 4 Halal Research Center of IRI, FDA, Tehran, Iran.; 5 Biotechnology Research Center, Pharmaceutical Technology Institute, Mashhad, Iran.; 6 Changshu Hospital Affiliated to Soochow University, Changshu No.1 People's Hospital, Changshu 215500, Jiangsu Province, China.

**Keywords:** GLP-1RAs, CVD, diabetes, CVOT, glycemic control, MACE

## Abstract

Type 2 diabetes mellitus (T2DM) is closely associated with cardiovascular diseases (CVD), including atherosclerosis, hypertension and heart failure. Some anti-diabetic medications are linked with an increased risk of weight gain or hypoglycemia which may reduce the efficacy of the intended anti-hyperglycemic effects of these therapies. The recently developed receptor agonists for glucagon-like peptide-1 (GLP-1RAs), stimulate insulin secretion and reduce glycated hemoglobin levels without having side effects such as weight gain and hypoglycemia. In addition, GLP1-RAs demonstrate numerous cardiovascular protective effects in subjects with or without diabetes. There have been several cardiovascular outcomes trials (CVOTs) involving GLP-1RAs, which have supported the overall cardiovascular benefits of these drugs. GLP1-RAs lower plasma lipid levels and lower blood pressure (BP), both of which contribute to a reduction of atherosclerosis and reduced CVD. GLP-1R is expressed in multiple cardiovascular cell types such as monocyte/macrophages, smooth muscle cells, endothelial cells, and cardiomyocytes. Recent studies have indicated that the protective properties against endothelial dysfunction, anti-inflammatory effects on macrophages and the anti-proliferative action on smooth muscle cells may contribute to atheroprotection through GLP-1R signaling. In the present review, we describe the cardiovascular effects and underlying molecular mechanisms of action of GLP-1RAs in CVOTs, animal models and cultured cells, and address how these findings have transformed our understanding of the pharmacotherapy of T2DM and the prevention of CVD.

## Introduction

Over 90% of diabetes is classified into two main types being Type 1 diabetes mellitus (T1DM) and T2DM. T1DM is defined as an autoimmune disease, resulting in insulin deficiency. T2DM is a metabolic disease caused by acquired endocrine disorders. T2DM is the underlying cause of many complications, among which, cardiovascular diseases (CVDs) are mainly responsible for the high rates of morbidity and mortality. Atherosclerotic CVD manifest as heart attacks and strokes, is responsible for up to 85 per cent of cardiovascular deaths 1. Atherosclerosis emerges as the “invisible killer” lurking in the cardiovascular system. With the gradual progression of atherosclerotic plaques, atherosclerosis will induce angina pectoris, or cerebral ischemia, leading to severe clinical manifestations such as heart failure, myocardial infarction (MI), cerebral infarction and lower limb ischemia and amputation 2.

Multi-country, multi-center long-term cardiovascular outcome trials (CVOTs) confirmed that GLP-1RAs decrease cardiovascular mortality and provide cardiovascular benefits to reduce the incidence of MI or non-fatal stroke 3. For example, in the LEADER trial, when compared with placebo, liraglutide decreased the incidence of major cardiovascular events (MACE) by 13%, cardiovascular death by 22% and all-cause death by 15% among T2DM patients with CVD, on a standard treatment basis 4. In addition, liraglutide provided multiple additional benefits by reducing the incidence of hypoglycemia, lowering HbA1c and functioning through multiple mechanisms to better control multiple causes of MACE, such as blood lipids, BP as well as body weight (BW).

GLP-1 is a gut-derived insulin agonist with the ability to suppress glucagon release and stimulate insulin secretion through targeting α-cells and β-cells, respectively 5. Likewise, GLP-1RAs can lower postprandial glycemia predominantly by slowing gastric emptying and promoting weight loss 6, 7. GLP-1RAs may improve endothelial cell function via anti-inflammatory and vasodilatory properties. Also, anti-proliferative effects on smooth muscle cells and anti-inflammatory effects on macrophages may contribute to the protection against the development and progression of atherosclerosis 8-10. In consideration of the dual roles of GLP-1RAs in hypoglycemic effects and preventing CVD among T2DM patients, we reviewed the cardiovascular effects and molecular mechanisms of GLP-1RAs in CVOTs, animal models and cultured cells, and discuss how these findings transform pharmacotherapies for T2DM, and even expand the indication of this important category of anti-diabetic drugs to CVD patients without diabetes.

## Clinical GLP-1RAs and their pharmacological basis of action

GLP-1RAs, consist of short-acting and long-acting agents. Short-acting drugs include exenatide twice daily, lixisenatide once daily and oral semaglutide once daily, while long-acting drugs include liraglutide, semaglutide, exenatide, albiglutide and dulaglutide all being administered once weekly. The primary pharmacodynamic distinctions between long-acting and short-acting GLP-1RAs are that long-acting agonists augment insulin production and suppress glucagon generation to lower postprandial glucose and fasting plasma glucose, while short-acting agonists chiefly lower postprandial glucose by delaying gastric emptying. Long-acting GLP-1RAs also have other benefits including improved gastrointestinal tolerability, moderate plasma drug concentration fluctuations, and more convenient dosage regimens which may improve medication adherence and treatment outcomes 11 (Table 1).

Exendin-4 was originally found in the saliva of the venomous lizards whose synthetic version is exenatide. Exendin-4 inhibits the remodeling in the remote myocardium of rats following acute MI by attenuating β-catenin activation and activating glycogen synthase kinase-3, β-arrestin-2 and protein phosphatase 2A 12. In addition, exendin-4 protects against cardiac ischemia/reperfusion injury in rats by enhancing antioxidant levels and inhibiting c-Jun NH2-terminal kinase (JNK)/p^66^ Shc/NADPH oxidase axis 13. GLP-1RAs, such as lixisenatide, not only rely on β-cell function and glucagon suppression, but also assist to lower glucose by other (insulin-independent) mechanisms such as delayed gastric emptying, underlying the clinical utility of GLP-1RAs as adjuvant therapy to basal insulin in longstanding T2DM 14. Further, liraglutide protects against vascular oxidative stress via attenuating eNOS (endothelial NO synthase) s-glutathionylation (a hallmark of eNOS uncoupling) and increasing NO bioavailability 15.

The GLP-1 analogs, semaglutide and liraglutide, delay the progression of atherosclerosis by regulating inflammatory pathways in low-density lipoprotein receptor-deficient (LDLr^-/-^) and apolipoprotein E-deficient (ApoE^-/-^) mice 16. Oral semaglutide reduces systolic blood pressure (SBP), BW and blood glucose levels. Nevertheless, it is associated with increased incidence of gastrointestinal adverse reactions 17. Exenatide ameliorates intramyocellular lipid deposition through improving insulin sensitivity and activating the AMP-activated protein kinase (AMPK) signaling pathway without BW reduction 18. Compared with twice-daily exenatide, dulaglutide treatment reduces glycated hemoglobin and fasting plasma glucose (FPG) to a greater extent 19. Taspoglutide prevents apoptosis *in vitro*, promotes β-cell proliferation, and exerts multiple protective effects of β-cell in Zucker Diabetic Fatty rats 20. Compared with both placebo and liraglutide at 26 weeks, orally bioavailable semaglutide is superior in reducing BW and non-inferior to liraglutide concerning for decreasing HbA1c 21.

Based on the above pharmacological characteristics of categories of GLP-1RAs agents, despite slight adverse gastrointestinal side effects, it is clear that GLP-1RAs agents have efficacy in cardiovascular protection without untoward hypoglycemia.

## Cardiovascular outcome trials of GLP-1RAs

In the past decade, CVOTs with GLP-1RAs have been conducted with at least seven agents including liraglutide, lixisenatide, exenatide, semaglutide, dulaglutide, albiglutide and oral semaglutide (Table 2). In CVOTs, liraglutide, semaglutide and dulaglutide demonstrated appreciable cardiovascular and renal protective effects, among which liraglutide had an appreciable effect on mitigating the risk of cardiovascular death 4, while semaglutide and dulaglutide conferred a risk reduction on non-fatal stroke 22, 23. Moreover, liraglutide also had effects on reducing the incidence of MI in high-risk patients for T2DM, and may improve the clinical outcomes of MI 24.

To date, the REWIND study has demonstrated for the first time that primary cardiovascular prevention strategies can significantly reduce cardiovascular adverse events in patients with T2DM, regardless of whether or not these patients are co-diagnosed with CVD 23. In this regard, dulaglutide is an ideal hypoglycemic drug for patients with T2DM due to its reported utility in primary prevention and secondary prevention. By February 21, 2020, dodecaplatin^®^ (dulaglutide) is the first and only FDA-approved anti-diabetic drug for primary and secondary prevention to alleviate the risk of MACE 25.

### Hyperglycemia

All five long-acting GLP-1RAs can reduce glucose levels and BW 4, 22, 23, 26, 27. In SUSTAIN 3, SUSTAIN 7 and SUSTAIN 10, the GLP-1RAs semaglutide reduced HbA1c by up to 1.8%, achieved the glycemic control target of HbA1c in 80% of the intervention group, and reduced BW by up to 6.5 kg, outcomes which were superior to liraglutide, exenatide and dulaglutide 28-30. In the LEAD-5 trial, liraglutide (1.8 mg once daily) reduced HbA1c more than insulin glargine during the treatment period (26 weeks) 31. The results of AWARD-CHN2 showed that the reduction of FPG by dulaglutide was similar to insulin glargine. In addition, post-prandial blood glucose reduction by dulaglutide was superior to insulin glargine. In addition to this, dulaglutide can greatly stabilize and decrease blood glucose levels 32.

### Atherosclerosis and coronary artery disease

The levels of HbA1c, dyslipidemia, BW and BP are several of the risk factors for the progression of atherosclerosis and CVD in patients with T2DM. Several clinical trials have demonstrated that most of GLP-1RAs improve these parameters and thus attenuate the development and progression of atherosclerosis, especially concerning low-density lipoprotein cholesterol (LDL-C) levels 33. In 2015, a randomized controlled trial showed that taspoglutide can reduce total cholesterol, LDL-C and triglycerides 34. Among new-onset patients with diabetes receiving standard statin therapy, liraglutide together with metformin improved lipid distribution of LDL and C-reactive protein (CRP) in atherosclerosis 35. Studies have also reported that under the conditions of comparable glycemic control, liraglutide (1.2 mg/d) alone was more effective than liraglutide with metformin or metformin alone on lipid metabolism and cardiovascular protection 36.

### Hypertension

In the LEAD series of studies, clinical data demonstrated that treatment with liraglutide for 26 weeks can reduce SBP 4. Exenatide improved the BP and blood lipid level of T2DM patients for 7 consecutive years in a DURATION open-extension study 37. In the REWIND study and AWARD5 study, dulaglutide consistently improved BP and lipid levels in patients 23, 38. Mechanistically, GLP-1 can lower BP through a diuretic mechanism, and other effects on the kidneys 39, 40. At another level, GLP-1RAs also improve systemic insulin sensitivity to lower arterial hypertension in T2DM by decreasing the concentration of angiotensin II (Ang II) 41, 42.

### Heart failure

There are two main trials (FIGHT and LIVE trials) demonstrating that treatment with liraglutide increased the risk of adverse heart failure-associated outcomes in patients with heart failure and reduced LVEF 43-45. In the LIVE trial, liraglutide treatment resulted in no change in left ventricular systolic function, whereas liraglutide related to an increase in heart rate and more serious cardiac adverse events among chronic heart failure patients with and without type 2 diabetes 45. While in a FIGHT trial, liraglutide reduced HbA1c levels (-0.48; 95% CI -0.92, -0.04; P = 0.033) and triglyceride levels compared with placebo (-33.1 mg/dL; 95% CI -60.7, -5.6; P = 0.019), together with a substantial weight reduction (-4.10 lbs; 95% CI -7.94, -0.25; P = 0.0367) 44. Curiously, LEADER revealed a downward trend in hospitalization rates for heart failure (-13%, P = 0.14) 4.

### Chronic kidney disease (CKD)

CKD is clinically characterized by a decline in glomerular filtration rate (GFR) and proteinuria for diabetic nephropathy, which is determined by the pathological changes in the structure and function in the kidney of patients with T2DM. Several recent CVOTs have shown that GLP-1RAs can reduce the decline of estimated GFR (eGFR) and delay the onset of the occurrence of proteinuria in patients with T2DM 4, 22, 26, 46, 47.

In the ELIXA trial, lixisenatide ameliorated macroalbuminuria assessed as a decline in the urinary albumin-to-creatinine ratio. An analysis of the ELIXA trial data indicated that short-term GLP-1RAs may also have renal protective effects, analogous to long-acting GLP-1RAs 47. Analysis of the LEADER trial data demonstrated that liraglutide exerted a 22% decline in the morbidity of newly diagnosed or aggravated nephropathy 4. While in SUSTAIN-6, a sharper decline (46%) in macroalbuminuria seems to be entirely due to the semaglutide treatment than a 26% fall in macroalbuminuria by liraglutide 22. The AWARD-7 trial, which was the first clinical trial among moderate-to-severe CKD and T2DM displayed convincing protective effects on eGFR after treatment with GLP-1RAs 46. In addition, the analysis of the EXSCEL trial revealed that a composite end-point of new macroalbuminuria, 40% eGFR decline or renal death was reduced by the addition of exenatide in T2DM 26.

To summarize several meta-analyses of CVOTs conducted with GLP-1RAs, it isconfirmed that based on 43 trials, GLP-1RAs are a class of efficacious agent for reducing MACE (MH-OR 0.87 [0.83, 0.92]), cardiovascular and all-cause mortality (MH-OR 0.89 [0.83, 0.96]) 48. Further, GLP-1RAs significantly reduce the risk of MI and stroke, but have a neutral effect on hospitalization for heart failure upon 5 available CVOTs 49. Meta-analysis of 7 CVOT pooled demonstrated that GLP-1RAs were associated with a similar decrease in MACE regardless of gender 50. From one hundred and thirteen available trials, the safety of GLP-1RAs was confirmed in pancreatitis. Conversely, treatment with GLP-1RAs can increase cholelithiasis mobility 51.

## Cardiovascular actions of GLP-1RAs in pre-clinical studies

### Effects of GLP-1RAs on atherosclerosis and atherosclerosis in the setting of diabetes

Atherosclerosis with multiple and diverse drivers is a dominant complicating process in T2DM 53. Exposure to western diets pathologically alters the expression of many genes leading to atherosclerosis 16. According to a recent study, GLP-1R is mainly expressed in the macrophage enrichment region of atherosclerotic plaques, which provide us a new perspective on pharmacological modification of GLP-1R upon signal in atherosclerosis 54.

Preclinical studies have shown that large doses of GLP-1RAs can reduce lipid deposition and plaque volume on the aortic surface of hyperglycemic mice via an AMPK-independent action in streptozotocin (STZ)-induced hyperglycemic and hyperlipidemic mice or type 1 diabetic rats 55. GLP-1RAs have direct anti-atherosclerotic effects in euglycemic mice, ApoE^-/-^ mice or Watanabe heritable hyperlipidemic (WHHL) rabbits in a GLP-1R-dependent manner 56-58. Therefore, these studies demonstrated that GLP-1RAs have an impact on preventing and stabilizing atherosclerotic vascular disease, which may translate into its protective effects against MACE in human subjects 59.

GLP-1RAs suppress the progression of atherosclerosis in ApoE^-/-^, LDLr^-/-^, and even in moderate uremia LDLr^-/-^ mice^16^, ^60^. This suppression is independent of plasma cholesterol changes and BW reduction through regulating markers of plaque instability and inflammation including those linked to plaque hemorrhage (CD163), matrix turnover (matrix metalloproteinase (MMP)-3, MMP-13), cholesterol metabolism (prostaglandin I2 synthase (PTGIS), ATP-binding cassette transporter 1 (ABCA1)), leukocyte recruitment (C-C motif chemokine ligand 2 (CCL2), osteopontin (OPN), interleukin (IL)-6), and leukocyte rolling, extravasation and adhesion (vascular cell adhesion molecule (VCAM)-1, E-selectin (SELE)) 16. Moreover, GLP-1RAs can alleviate vascular remodeling expressed as neointimal hyperplasia after induced vascular injury. Specifically, GLP-1RAs can suppress vascular smooth muscle cell (VSMC) proliferation and migration via the cAMP/PKA pathway in C57BL/6 mice 61. These findings indicate that GLP-1RAs limit and stabilize the development of atherosclerotic plaques through anti-inflammatory mechanisms and by preventing vascular remodeling 62.

**Figure 1:**
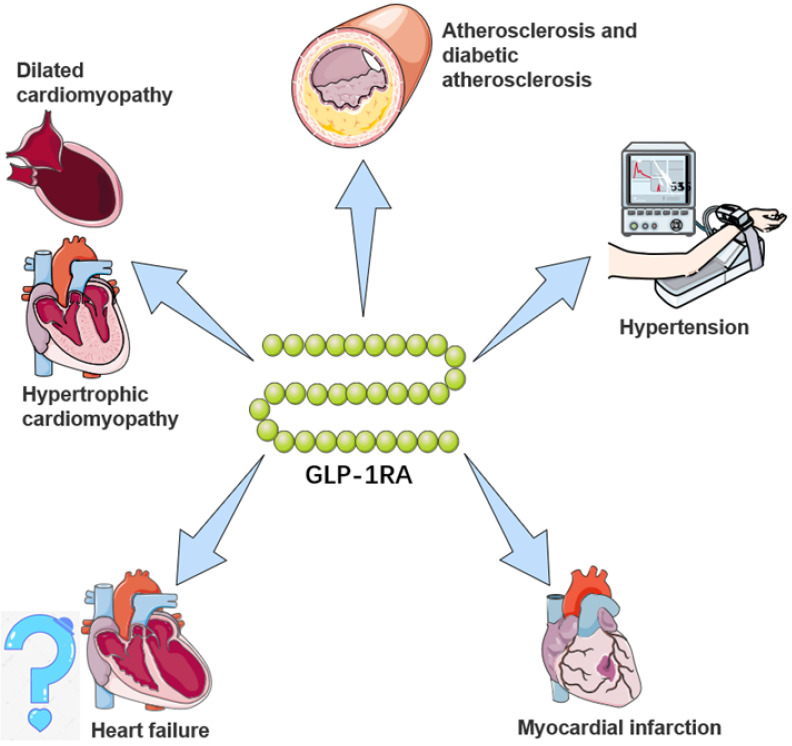
**The potential cardiovascular benefits of GLP-1RA.** GLP-1RAs have effects on limiting atherosclerosis, controlling hypertension, and delaying the progression of heart failure, myocardial infarction, and cardiomyopathy. For a brief description, GLP-1RAs attenuate and stabilize the development of atherosclerotic plaques through anti-inflammatory mechanisms and preventing vascular remodeling, and inhibit activation and recruitment of macrophages in atherosclerosis. For hypertension, GLP-1RAs augment renal blood flow and promote natriuresis and diuresis. For myocardial infarction, GLP-1RAs alleviate and repair MI via SIRT1/Parkin/mitophagy, insulin-like growth factor-1/2 and upregulating α-estrogen receptor. For heart failure, GLP-1RAs remodel calcium circulation disorders, inhibit cardiac hypertrophy by activating the AMPK/ mTOR signaling pathway and attenuate methylglyoxal-induced mitochondrial abnormalities. For cardiomyopathy, GLP-1RAs improve mitochondrial function via regulating autophagy and inflammatory signaling, as well as not only mediate the inhibition of myocardial apoptosis, but also improve cardiac energy metabolism.

GLP-1RAs attenuate the activation and recruitment of macrophages into the endothelium 63. Treatment with GLP-1RAs can reduce monocyte/macrophage accumulation in the arterial wall by inhibiting the adhesion of monocytes to activated endothelium from the arteries of C57BL/6 mice as well as in ApoE^-/-^ mice 64. GLP-1RAs can also exert anti-inflammatory effects on macrophages as well as regulating macrophage phenotype 65. For example, treatment of murine macrophages with GLP-1RAs *in vitro* inhibits monocyte chemoattractant protein-1 gene expression and p65 as well as tumor necrosis factor (TNF)-α expression. Moreover, GLP-1RAs can suppress atherosclerosis in T2DM (STZ treated) rats by inhibiting macrophage infiltration and apoptosis 66. The above studies suggest that GLP-1RAs may be a valuable strategy for the treatment of atherosclerosis (Figure 1) (Table 3).

### Effects of GLP-1RAs on hypertension

Hypertension and accompanying cardiovascular remodeling are amongst the leading drivers of atherosclerosis and causes of CVD. Endogenous GLP-1R signaling exerts a physiologically relevant effect on BP control, which may be attributable, in part, to its tonic actions on proximal tubule Na/H mediated sodium reabsorption, and the intrarenal renin-angiotensin II system 67. In spontaneously hypertensive rats, GLP-1 treatment mediates renal vasodilation and attenuates renal autoregulatory responses 68. In addition, acute renal infusion with GLP-1 can augment renal blood flow and promote natriuresis and dieresis 69. In summary, GLP-1 or GLP-1RA treatment can make a positive impact on reducing the impact of hypertension in affected patients (Figure 1).

### Effects of GLP-1RAs on myocardial infarction (MI)

MI and its consequent tissue damage leading to heart failure are mainly responsible for the fatality in patients with T2DM. Evidence indicates that GLP-1RAs can reduce the occurrence of MI 70. Among patients with acute MI undergoing percutaneous interventions, GLP-1 treatment can reduce infarct size, together with improving left ventricular ejection fraction (LVEF) 71. GLP-1RA (such as exendin-4) treatment can reduce the size of MI impacted cardiac volume in rats, inhibit ventricular dilation, myocardial fibrosis and hypertrophy *in vivo* 72. Mechanically, GLP-1RAs regulate insulin-like growth factor-1/2 and upregulate α-estrogen receptors to exert cardioprotective actions in isoprenaline-induced MI 73. In addition, another report demonstrated the reparative role of GLP-1RAs in MI via modulating sirtulin (SIRT)1/Parkin/mitophagy 74. Interestingly, even in obese swine, GLP-1R activation can improve cardiac function and augment cardiac output after MI 75. Accordingly, it is suggested that GLP-1RAs have properties of alleviating MI and repairing damaged cardiac tissue to improve cardiac function (Figure 1).

### Effects of GLP-1RAs on heart failure

Myocardial remodeling has a critical role in heart failure following MI. Disturbances in cardiac calcium metabolism are a hallmark of heart failure. GLP-1RAs prevent post-MI remodeling by having an impact on the extracellular matrix changes rather than dependency on its glycemic control 76. There is another mechanism showing that GLP-1RA treatment regulates Ca^2+^ levels due to reductions in the ryanodine receptor (RyR)2 phosphorylation and suppression of the activation of calmodulin-dependent protein kinase (CaMK)-II 77. Moreover, a favorable impact of GLP-1RAs on cardiac remodeling can occur through activation of the eNOS/cyclic guanosine monophosphate (cGMP)/protein kinase G (PKG) nexus 72.

Cardiac hypertrophy leads to heart failure at the later decompensated stage of the disease process, which is a primary cause of morbidity and mortality. Evidence has recently revealed that GLP-1RAs inhibit cardiac hypertrophy by upregulating GLP-1R expression and activating the AMPK/mechanistic target of the rapamycin (mTOR) signaling pathway 78.

Mitochondrial dysfunction, apoptosis and oxidative stress are in part due to methylglyoxal accumulation. Therefore, the attenuation of methylglyoxal-induced mitochondrial abnormalities may suppress the progression of heart failure. In cultured H9c2 cells, GLP-1RAs attenuate the production of mitochondrial and intracellular reactive oxygen species (ROS) induced by methylglyoxal. As a result, GLP-1RAs display suppressive effects on mitochondrial functions and oxidative stress on heart failure 79.

Although GLP-1RAs can play a favorable role in improving heart failure based on pre-clinical studies, whereas some clinical evidence relevantly have reported that GLP-1RAs treatment could be associated with adverse heart failure events, eg. increasing heart rate, reducing LVEF 43-45. Therefore, overall, the benefit of GLP-1RAs for heart failure has been difficult to confirm (Figure 1).

### Effects of GLP-1RAs on cardiomyopathy

Diabetic cardiomyopathy is an important cardiac complication which is accompanied by cardiac hypertrophy 78. Chronic inflammation results in the loss of cardiomyocytes in diabetic hearts. GLP-1RAs have the potential to inhibit cardiac inflammation via retarding the production of pyroptotic cytokines, caspase-1 and the AMPK-thioredoxin interacting protein (TXNIP) pathway in high-fat diet-fed rats 80. Specifically, GLP-1RAs exert protective effects on exercise-induced cardiomyopathy via increasing autophagy, reducing inflammation-related proteins and ROS generation via restoring the expression of catalase and manganese superoxide dismutase (MnSOD) 81.

GLP-1RAs attenuate apoptosis in cardiomyocytes induced by hyperglycemia via inhibiting receptor of advanced glycation end products (RAGE) receptor 82. In H9c2 cardiomyoblasts, GLP-1RAs protect against cell apoptosis and inhibit the expression of tumor protein P53 (p53). GLP-1RAs not only mediate the inhibition of myocardial apoptosis, but also improve cardiac energy metabolism. These studies illustrate that GLP-1RAs may confer protective effects on the progression of cardiomyopathy 83.

GLP-1RA treatment also mitigates experimental diabetic cardiomyopathy by the indirect mechanisms of augmenting glucose oxidation in cardiomyocytes 84. In terms of mechanisms, GLP-1RAs can block C/EBP homologous protein (CHOP)-mediated endoplasmic reticulum (ER) stress via attenuating the irreversible electroporation (IRE)-α unfolded protein response (UPR) 85. There is a further action that the reduction of HOX transcript antisense RNA (HOTAIR) expression gives rise to cardiomyopathy. As a result, GLP-1RAs demonstrate a new therapeutic action in cirrhotic rats with cardiomyopathy through the protective role of HOTAIR via SIRT1 activation 86.

Abnormal autophagy and mitochondrial injury are involved in diabetic cardiomyopathy. The cardioprotective effects of mesenchymal stem cells, GLP-1RAs or pioglitazone may improve mitochondrial function via regulating autophagy and inflammatory signaling 87. Several studies revealed that GLP-1RAs relieve myocardial damage and glucose toxicity by promoting autophagy, which is associated with decreasing mTOR phosphorylation and increasing AMPK phosphorylation 88. Furthermore, a new orally bioavailable GLP-1RA, oral hypoglycemic peptide 2 (OHP2), can confer protective effects on diabetic cardiomyopathy through rescuing cardiac lipotoxicity by inhibiting Rho (the small GTPase) kinase (ROCK)/PPARα pathway 89, 90 (Figure 1).

## Molecular mechanisms of GLP-1RA-mediated cardiovascular actions

### Lowering blood glucose levels by enhancing insulin secretion

T2DM is characterized by the presence of chronic fluctuating hyperglycemia, obesity-associated insulin resistance as well as the impaired secretion of insulin and dysfunction of the incretin system. Although endogenous GLP-1 lowers blood glucose levels, it is degraded quickly, which results in loss of its hypoglycemic effects. It follows that long-acting GLP-1RAs have a longer half-life and show prolongation of the glucose-lowering effects 3.

**Figure 2:**
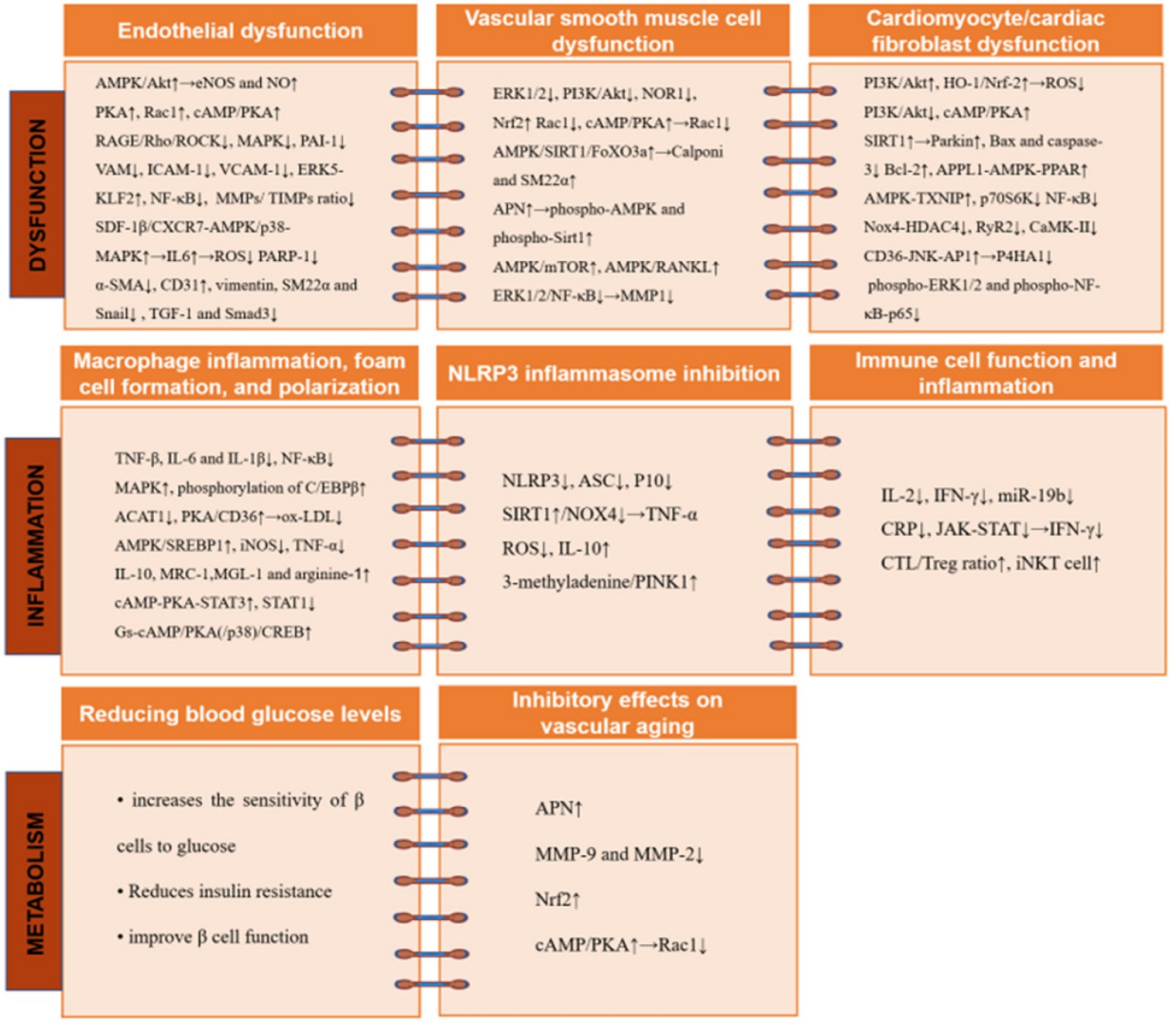
** The cardiovascular protective effects of GLP-1RA involve multiple molecular mechanisms and signaling pathways.** GLP-1RA inhibit endothelial cell dysfunction while attenuating abnormal migration, proliferation, and apoptosis in VSMCs. In addition, GLP-1RA decrease macrophage inflammation and blocks NLRP3 inflammasome activation. Further, GLP-1RA protect against vascular aging and maintains the metabolic homeostasis of cardiomyocytes. Abbreviations: ACAT: acyl-CoA cholesterol acyltransferase; AMPK: AMP-activated protein kinase; APN: adiponectin; APPL: activating the leucine zipper motif'; ASC: apoptotic speck containing protein; Bax: Bcl-2-associated x; Bcl: B-cell lymphoma; CaMK: calmodulin-dependent protein kinase; cAMP: cyclic adenosine monophosphate; CD31: cell adhesion molecule; C/EBP β: CCAAT/enhancer-binding protein β; CREB: cAMP response element binding-protein; CRP: C-reactive protein; CTL: cytotoxic T lymphocyte; CXCR: C-X-C motif receptor; eNOS: endothelial NO synthase; ERK: extracellular signal-regulated kinase; FOXO: forkhead box O; HDAC4: histone deacetylase 4; HO-1: heme oxygenase-1; ICAM-1: intracellular adhesion molecule-1; IFN: interferon; IL: interleukin; iNKT: invariant natural killer T; iNOS: inducible NOS; JAK: Janus kinase; JNL: Jun NH2-terminal kinase; KLF2: Kruppel-like factor 2; MAPK: mitogen-activated protein kinases; MGL-1: macrophage galectin-1; MMP: matrix metalloproteinase; MRC-1: mannose receptor-1; mTOR: mechanistic target of rapamycin; NF-κB: a nuclear factor-κB; NLRP3: Nod-like receptor protein 3; NOR1: neuron-derived orphan receptor 1; Nox4: NADPH oxidase 4; Nrf2: nuclear factor erythroid 2-related factor 2; ox-LDL: oxidized-LDL; PAI: plasminogen activator inhibitor; PARP-1: poly(ADP-ribose) polymerase 1; PINK1: the mitochondrial kinase; PI3K: phosphoinositide 3-kinase; PKA: protein kinase A; PPAR: peroxisome proliferator-activated receptor; P10: cleaved caspase 1; P4HA1: prolyl 4-hydroxylase subunit alpha-1; p70S6K: p70 ribosomal protein S6 kinase; Rac1: Ras-related C3 botulinum toxin substrate 1; RAGE: receptor AGE; RANKL: receptor activator of nuclear factor kappa B ligand; Rho: the small GTPase; ROCK: Rho kinase; ROS: reactive oxygen species; RyR2: the type 2 ryanodine receptor; SDF: stromal cell-derived factor; SREBP1: element binding transcription factor 1; SIRT: sirtulin; α-SMA: alpha smooth muscle actin; SM22α: sensitive 22 kDa actin-binding protein of the calponin; STAT: cAMP-PKA-signal transducers and activators of transcription; TGF: transforming growth factor; TIMP: tissue inhibitor of MPs; TNF: tumor necrosis factor; Treg: regulatory T cell; TXNIP: AMPK-Thioredoxin-interacting protein; VAM: vascular adhesion molecule; VCAM-1: vascular cell adhesion molecule 1; VSMC: vascular smooth muscle cells.

GLP-1RAs can improve β cell function in patients with early T2DM via coordinated regulation of the fate of α and β cells. There is evidence showing that GLP-1RAs can reduce the expression of β cell apoptotic markers Bcl-2-associated x (Bax) and caspase-3 while upregulating Bcl-2 99, 100, as well as protecting against lipotoxic stress via phosphoinositide 3-kinase (PI3K)/Akt/forkhead box proteins O1 (FoxO1) pathway 101. GLP-1RAs increase the sensitivity of β cells to glucose and positively reduce insulin resistance to further promote insulin secretion 102. GLP-1RAs stimulate insulin secretion and suppress glucagon secretion, which may modify cardiac energy metabolism in T2DM patients 103. Based on the above mechanisms of β cells, GLP-1RA treatment exerts favorable effects on hyperglycemia (Figure 2).

### Reversing endothelial dysfunction by GLP-1RAs

The cardiovascular phenomenon of endothelial dysfunction involves a series of structural and functional abnormalities, including abnormal secretory capacity and properties of the endothelium and impaired vascular tone, as well as an impaired endothelial barrier function. GLP-1RAs stimulate NO production and eNOS activation, eliciting vasorelaxation in arterial endothelium *in vitro* 104. GLP-1RAs make a direct protective impact on the endothelium by the GLP-1R-dependent AMPK/Akt/eNOS pathway 105. GLP-1RAs also promote endothelial barrier integrity via activating protein kinase A (PKA)- and Ras-related C3 botulinum toxin substrate 1 (Rac1) 106. Moreover, GLP-1 exerts stabilizing effects on the endothelial contraction and barrier for AGE-treated endothelium via activating GLP-1R/cyclic adenosine monophosphate (cAMP)/PKA and inhibiting mitogen-activated protein kinase (MAPK) signaling pathways together with RAGE/Rho/ROCK 97, 98. Through the above mechanisms, GLP-1RAs can mediate the function of regulating endothelial-mediated contraction and vasodilation, as well as maintaining the stability of the endothelial barrier.

GLP-1RAs can improve the efficacy of protection against the adhesion of inflammatory cells to the endothelium. Specifically, GLP-1RAs downregulate activation of VCAM‑1, the intracellular adhesion molecule-1 (ICAM-1) and plasminogen activator inhibitor type 1 (PAI-1) in endothelial cells 57. Further, GLP-1RAs block the adhesion of monocytes to endothelium induced by oxidized LDL via extracellular signal-regulated kinase (ERK) 5-Kruppel-like factor (KLF)2 signaling pathway to prevent endothelial permeability 107, 108. Likewise, GLP-RAs inhibit platelet aggregation and prevent thrombosis 109. These findings indicate that GLP-1RAs have anti-adhesive and anti-thrombotic properties in the endothelium that may delay the development of atherosclerosis 110.

GLP-1RAs play a key role in regulating the response to inflammation of the endothelium. Specifically, GLP-1RAs block NLRP3 (Nod-like receptor protein 3h) inflammasome via SIRT1 activation 111. GLP-1RAs also suppress the nuclear factor-κB (NF-κB) pathway and free fatty acid-induced cellular ROS in endothelium 112. GLP-1RAs also downregulate the expression of MMPs and tissue inhibitors of MPs (TIMPs) for anti-inflammation 110. Furthermore, noted that GLP-1RAs can increase cholesterol excretion and reduce cholesterol accumulation by promoting the expression of ATP-binding cassette transporter A1 (ABCA1) in the endothelium. Therefore, treatment with GLP-1RAs shows favorable effects on anti-inflammation through the suppression of these proinflammatory cytokine and inflammatory signal pathways, as well as augmenting cholesterol efflux in the endothelium.

GLP-1RAs can exert not only anti-inflammatory effects in the endothelium, but also anti-apoptotic effects aimed at maintaining endothelial homeostasis. GLP-1RAs ameliorate ROS-induced or hyperglycemia-induced apoptosis through modulating IL-6 production and ER stress-induced by hyperhomocysteinemia, via stromal cell-derived factor (SDF)-1β/C-X-C motif receptor 7 (CXCR7) -AMPK/p38-MAPK axis 113-115. Hereby, the mechanisms of the anti-apoptotic actions involve not only protein kinase cascades, but also the modulation of miRNA levels. Treatment with GLP-1RAs can regulate several pivotal miRNAs (such as miR-93-5p, miR-181a-5p and miR-34a-5p and miR-26a-5p) to inhibit apoptosis in endothelium 116. These findings collectively support the anti-apoptotic actions of GLP-1RAs as part of their effects on endothelial dysfunction.

Under high glucose conditions, endothelial cells acquire the characteristics of fibroblasts, via endothelial-mesenchymal transition (EndMT). EndMT promotes diabetic cardiac fibrosis 117. EndMT relies on activating the Smad2/3-Slug pathway at an early stage 118. GLP-1 treatment protects against EndMT through inhibiting the activation of poly (ADP-ribose) polymerase 1 (PARP-1) which can elicit Smad3 and transforming growth factor (TGF)-β1 responses. Moreover, GLP-1RAs can reverse EndMT induced by high glucose and IL-1β via activating the AMPK pathway, which was evidenced by augmenting an endothelium marker expression (CD31), as well as alleviating mesenchymal markers expression (SM22α (sensitive 22 kDa actin-binding protein of the calponin), vimentin and Snail) 119. These results indicate that GLP‑1RAs have therapeutic effects against EndMT, an important process in atherosclerosis and fibrotic diseases.

To summarize these mechanisms and their corresponding efficacy with GLP-1RAs or GLP-1, GLP-1RAs show the endothelial protective properties and thus owe to modulating vascular tone and maintaining endothelial barrier integrity, as well as the anti-adhesive and anti-thrombotic actions. In addition, the inhibition of EndMT, the attenuation of inflammation and apoptosis are also responsible for reversing endothelial dysfunction with GLP-1RA treatment (Figure 2).

### Improving vascular smooth muscle cell (VSMC) dysfunction by GLP-1RAs

VSMC dysfunction can be defined as the occurrence of excessive proliferation, autophagy, fibrosis, senescence, and phenotypic transformation. Accordingly, VSMC dysfunction ultimately aggravates vascular injury and accelerates the development of atherosclerosis. GLP-1RAs suppress hyperglycemia-induced proliferation, migration and apoptosis of VSMCs via suppressing the ERK1/2 and PI3K/Akt pathways 10. In terms of proliferation, liraglutide attenuates VSMC proliferation induced by Ang II through eliciting cell cycle arrest and activating AMPK 120. Furthermore, the number of neuron-derived orphan receptor 1 (NOR1) which can modulate VSMC proliferation, is decreased by GLP-1RAs in a dose-dependent manner in mice 121.

VSMC senescence induced by Ang II is another important aspect of VSMC dysfunction. In this regard, GLP-1RAs can provide the protection against VSMC senescence via increasing the nuclear factor erythroid 2-related factor 2 (Nrf2) activity 122. In addition, GLP-1RAs inhibit superoxide anion formation and ensuing VSMC senescence through suppressing Rac1 activation via cAMP/PKA pathways 123.

VSMC phenotypic switching is characterized by transformation from a quiescent contractile to an activated synthetic phenotype, promoting proliferation and leading to the formation of the neointima, the pathological vascular compartment critical for the development of atherosclerosis. In rat coronary artery smooth muscle cells, GLP-1RAs can inhibit phenotypic modulation induced by AGEs through blocking the NF-κB signaling pathway 124. Further, GLP-1RAs promote VSMC redifferentiation via AMPK/SIRT1/forkhead box O (FOXO3a) pathways, where the expression of contractile VSMCs markers (SM22α and Calponin) is increased 125. Likewise, GLP-1 inhibits VSMC dedifferentiation by regulating mitochondrial dynamics via enhancing adiponectin (APN) expression, which in turn promotes the phosphorylation of AMPK and SIRT1 in VSMC 126, 127.

Osteoblastic differentiation of VSMCs plays an important cytopathological role in arterial calcification. By inhibiting osteoblast differentiation of VSMCs, GLP-1RAs can ameliorate phosphate-induced vascular calcification, which is mediated by the AMPK/mTOR pathway or AMPK/receptor activator of nuclear factor kappa B ligand (RANKL), as occurs in the osteoblast differentiation of VSMCs 128, 129.

Arterial restenosis and vascular remodeling are a response to vascular injury, usually manifested as neointimal hyperplasia. GLP-1RAs can inhibit neointimal hyperplasia by suppressing VSMC proliferation and migration as mentioned above. GLP-1 treatment can exert the direct action on reversing vascular remodeling by downregulating MMP1 expression via alleviating the ERK1/2/NF-κB pathway in Ang II-induced rat aortic VSMCs 130. Furthermore, GLP-1R activation can prevent arterial restenosis through inhibiting VSMC proliferation and migration via stress-associated endoplasmic reticulum protein 1 (SERP1) 131.

As a result, GL-1RAs play a role in reversing or improving VSMC dysfunction by suppressing VSMCs proliferation, migration, apoptosis, as well as inhibiting VSMC phenotypic switching, especially inhibiting osteoblastic differentiation. GLP-1RAs have an impact in reversing vascular remodeling and attenuating the progression of VSMC senescence (Figure 2).

### Reducing macrophage inflammation, foam cell formation, and polarization by GLP-1RAs

Under pathological stress, macrophages contribute to inflammatory responses, which drive insulin resistance and T2DM. In this regard, GLP-1RAs (exendin-4, for example) not only alleviate macrophage infiltration, but also suppress IL-1β, IL-6 and TNF-α expression. Mechanistically, in macrophages, GLP-1RAs directly ameliorate NF-κB activation, thereby improving insulin resistance 132. In addition, GLP-1RAs and metformin can suppress pro-inflammatory phenotypes of monocyte/macrophages through impacts on the CCAAT/enhancer-binding protein beta (C/EBP β), together with MAPK and NF-κB 133. As a result, GLP-1RAs can inhibit macrophage‑associated inflammation through the above mechanisms.

The transformation of monocyte/macrophages into foam cells, is the hallmark of atherogenesis. Vascular located monocytes/macrophages engulf oxidized-LDL (ox-LDL), generating foam cells which release a plethora of pro-inflammatory mediators. The process of foam cell formation occurs via the imbalanced influx and efflux of modified lipoproteins. After lipid uptake, acyl-CoA: cholesterol acyltransferase 1 (ACAT1) catalyzes cholesterol esterification. GLP-1RAs can suppress foam cell formation by activating GLP-1R signaling induced autophagy 134, reducing ACAT1 expression/activity 93, as well as inhibiting the PKA/CD36 pathway to inhibit ox-LDL uptake 135. Although in foam cells, GLP-1RAs also can protect against oxidative stress and steatosis via modulation of AMPK/sterol regulatory element binding transcription factor 1 (SREBP1) 136. Therefore, the reduction of foam cells with GLP-1RAs treatment is speculated to inhibit vascular senescence and delay the progression of atherosclerosis.

The activation of macrophages by GLP-1 also contributes to anti-inflammatory factor secretion and M2 polarization. In this regard, GLP-1 treatment leads to decreased expression of macrophage‑specific markers for M1 (inducible NOS (iNOS), IL‑6 and TNF‑α) and enhances expression level of M2-marker genes, including arginine‑1, macrophage galectin-1 (MGL-1), IL‑10, as well as mannose receptor-1 (MRC-1). Mechanistic evidence has indicated that GLP-1RAs promote bone marrow-derived macrophage polarization into the M2 phenotype via the cAMP-PKA-signal transducers and activators of the transcription 3 (STAT3) pathway 137, 138. It is acknowledged that activation of STAT3 which is crucial to M2 phenotype transformation, while STAT1 activity which is a key determinant for the M1 subtype. Hereby, GLP-1RAs can diminish STAT1 activity 91. By modulating macrophage polarization towards an M2 subtype, GLP-1RAs can inhibit inflammation occurring in the vessel lumen, an action which would alleviate the progression of atherosclerosis (Figure 2).

### Inhibition of NLRP3 inflammasome by GLP-1RAs

NLRP3 inflammasome activation induced by hyperglycemia can cause endothelial dysfunction in T2DM. GLP-1RAs suppress NLRP3 inflammasome activation via attenuating the expression of NLRP3, apoptotic speck containing protein (ASC), and cleaves caspase 1 (p10) 111. Besides, in H9c2 cardiomyocytes, GLP-1RAs also inhibit hypoxia-induced NLRP3 inflammasome activity and TNF-α to attenuate pyroptosis through modulating the SIRT1/NADPH oxidase 4 (NOX4)/ROS pathway 139. Whereas, the suppression of NLRP3 can be functioned by GLP-1RAs not only directly but also indirectly.

In addition, the inhibition of NLRP3 activation by GLP-1RAs requires regulation of the mitochondrial pathway. GLP-1RAs augment the mitophagy pathway to suppress NLRP3 inflammasome and ameliorate oxidative stress-induced injury in the liver 140. Hereby, the suppression of inflammatory injury with GLP-1RAs treatment can be eliminated by 3-methyladenine/the mitochondrial kinase (PINK1) siRNA 141. Moreover, what is clinically noteworthy, is that GLP-1RA treatment can reduce intimal hyperplasia after stent implantation by modulating the above pathway 142. Combined with above-mentioned data, GLP-1RAs show a positive impact on the suppression of NLRP3 inflammasome (Figure 2).

### Modulating immune cell function and inflammation by GLP-1RAs

The development of diabetes and obesity is underpinned by chronic inflammation 143. In this context, GLP-1RAs can inhibit the secretion of IL-2 and interferon (IFN)-γ, thereby regulating the immune microenvironment and preventing diabetes 144. GLP-1RA can inhibit the progression of atherosclerosis in the early phase by regulation of immune cell phenotypes. Particularly, liraglutide attenuates pre-established atherosclerosis throughout the aorta and aortic root in ApoE^-/-^ mice via increasing macrophage polarization into anti-inflammatory macrophages and by reducing the number of pro-inflammatory macrophages 92. By doing so, in atherosclerotic plaques from murine aortas, GLP-1RAs regulate proinflammatory mediators and the phenotype of immune cells.

The immuno-modulatory effects of GLP-1RAs also suggest their potential in T1DM. In non-obese diabetic (NOD) mice which are a murine model of T1DM, GLP-1RAs can improve immune functions and inflammatory responses as well as mitigating against islet cell damage, an action which may be related to the decrease in the expression of miR-19b 145. Similarly, GLP-1RAs exert the potential protection against inflammatory responses in T1DM through decreasing IFN-γ secretion and downregulating PD-1 expression in T cells by attenuating the Janus kinase (JAK)-STAT pathway 146, 147. Thus, GLP-1RAs play an underlying role in immune-regulation in T1DM (Figure 2).

### Inhibitory effects on vascular aging by GLP-1RAs

Aging is a crucial component of the pathogenesis of CVD, as reflected by increased oxidative stress and impaired antioxidant defense, responses which may be induced by psychosocial stress 122, 148. ROS and inflammation are main mediators of vascular senescence. GLP-1RAs alleviate vascular aging under chronic stress via increasing adiponectin (APN) levels and inhibiting aortic MMP-9 and MMP-2 expression in ApoE^-/-^ mice 96, 149. Excessive autophagy is another cause of vascular aging. GLP-1 treatment can attenuate abnormal autophagy induced by ROS and inflammation through upregulating HDAC6 via the GLP-1R-ERK1/2 pathway 150. ER stress and DNA damage are also responsible for vascular senescence. Regarding this, GLP-1RAs inhibit ER stress and promote protein folding via endoplasmic reticulum oxidoreductase (ERO1α) in an AMPK-dependent manner, as well as mediating the process of mitochondrial fusion 115, 151. For senescence of VSMCs, GLP-1RAs activate Nrf2 and inhibit Rac1 through cAMP/PKA pathways to prevent Ag II-induced senescence 122, 123. In brief, all of the mechanisms and actions that have protective efficiency on endothelium and VSMCs, can play a role in preventing vascular senescence (Figure 2).

### Improving cardiomyocyte/cardiac fibroblast dysfunction by GLP-1RAs

Cardiomyocyte/cardiac fibroblast dysfunction is related to abnormal proliferation, apoptosis, hypertrophy, as well as inflammation and fibrosis, which may be caused by mitochondrial dysfunction and metabolic disorders. Persistent hyperglycemia can mediate hypoxic or ischemic injury, as well as elicit cardiomyocyte contractile dysfunction and the NF-κB signaling pathway activation, thus inducing ER stress 152. GLP-1RA can help maintain a balance of energy metabolism in cardiomyocytes through activating PI3K/Akt as well as p38 MAPK pathways as well as activating Nrf-2/heme oxygenase-1 (HO-1), contributing to augmented adenosine triphosphate formation and glucose ingestion by cardiomyocytes 153-155. Based on these pathways, GLP-1RAs improve metabolic disorders to alleviate cardiomyocyte dysfunction.

Apoptosis of cardiomyocytes is usually irreversible, leading to myocardial damage. GLP-1RAs make an impact on anti-apoptosis through multiple pathways. Firstly, the impact is partly due to ameliorating caspase-3 and Bax activity and upregulating Bcl-2 156. Secondly, GLP-1RAs attenuate apoptosis induced by TNF-α by ameliorating mitochondrial dysfunction related to activating the GLP-1R/cAMP/PKA and AMPK/SIRT1 pathway 94, 157, 158. Thirdly, GLP-1RAs exert anti-apoptotic actions attributable to the activation of Notch signaling 159. Fourthly, GLP-1 protects cardiomyocytes from oxidative stress and apoptosis in diabetes mellitus, through the mTOR complex1/p70 ribosomal protein S6 kinase (p70S6K) pathway 160. Fifth, GLP-1RAs protect against cardiomyocyte apoptosis under diabetic conditions via activating the leucine zipper motif (APPL_1_)-AMPK-PPARα axis and the AMPK-TXNIP pathway 80, 161.

Cardiac inflammation, fibrosis and hypertrophy are the main manifestations of myocardial dysfunction. Hyperglycemia-mediated cardiomyocyte damage is associated with inflammation. GLP-1RAs can have effects on inflammation and defending ER stress via suppressing NF-κB in diabetic cardiomyocyte models 152. GLP-1R can also suppress cardiac hypertrophy induced by Ang II through attenuating the Nox4-histone deacetylase 4 (HDAC4) axis 162. Apart from these actions, GLP-1RAs ameliorate cardiac fibrosis induced by abdominal aortic constriction via interfering with Ang II type 1 receptor signaling 163. Therefore, GLP-1RAs can provide protective effects on the above undesirable physiological phenomena in cardiomyocytes.

GLP-1RAs can modulate intracellular calcium homeostasis to prevent reperfusion injury directly in cardiomyocytes 164. GLP-1RAs also exert anti-arrhythmic effects via reducing calcium leak from the sarcoplasmic reticulum in ventricular arrhythmia, which has the potential to decrease phosphorylation of the type 2 ryanodine receptor (RyR2) and alleviate the activity of CaMK-II 77. Moreover, GLP-1R activation counteracts the effects of β-adrenoceptor stimulation on cardiac ventricular excitability and reduces ventricular arrhythmic potential 165. Apart from maintaining calcium channel homeostasis, GLP-1RAs also activate ATP-sensitive potassium channels to attenuate cardiac apoptosis and hypertrophy induced by pressure overload 166.

In addition to regulating cardiomyocyte function, GLP-1RAs also improve the function of cardiac fibroblasts. For example, GLP-1RAs may modulate the CD36-JNK-AP1 pathway by partially down-regulating prolyl 4-hydroxylase subunit alpha-1 (P4HA1), and then inhibit ROS production mediated by Ang II type I receptor, thereby ameliorating cardiac fibroblast proliferation and myocardial fibrosis 167, 168. Furthermore, as for cardiac fibroblasts, GLP-1RAs inhibit Ang II and glucose-induced collagen formation via decreasing phospho-NF-κB-p65 and phospho-ERK1/2 expression 169 (Figure 2).

To sum up, GLP-1RAs improve cardiomyocyte/cardiac fibroblast dysfunction by making balancing energy metabolism, and multiple pathways related to AMPK to inhibit cardiac apoptosis. Moreover, the suppression of cardiac inflammation, fibrosis and hypertrophy with GLP-1RA treatment provide a clinically favorable impact on cardiac dysfunction.

## Discussion

### Heterogeneous effects of GLP-1RAs in CVOTs

In the GLP-1RA family of agents, the results of CVOT have been inconsistent. The baseline characteristics of the study populations, study drugs and study designs may be considered as possible reasons for the variability 170. Furthermore, a real-world retrospective study comparing the compliance of patients receiving different GLP-1RAs for the treatment of hyperglycemic found that even if GLP-1RA weekly preparations were used, there were still differences in compliance 171. Such differences in compliance between GLP-1RA preparations are mainly due to the differences between injection devices 172. Ultimately, the duration of GLP-1RA treatment seems to be the factor that explains the heterogeneity of the clinical studies involving GLP-1RA.

Although some promising observations have been reported in various animal models, the impact of GLP-1RAs on human myocardial function is more heterogeneous. In a randomized controlled study, exenatide administered once a week for 18 months did not change carotid plaque volume or composition 173. Further, while GLP-1RAs have a positive impact on the LVEF, it seems to be inconsistent in most patients with heart failure and effects are quite limited 174. However, it is currently unclear and to what extent the differences observed in CVOT reflect the heterogeneous pharmacological properties of each drug in the GLP-1RA category. In order to enhance the clinical interpretation of these CVOTs, researchers have quantified the absolute treatment effect over time based on the number of treatments required to avoid a MACE, thus revealed the difference between GLP-1RA 175. Also, due to the important role of endothelial dysfunction in CVD, the effects of GLP-1RA on flow-mediated vasodilation (FMD) in patients remain to be investigated for their contribution to the CVD actions of GLP-1RAs.

### New pharmacological effects and molecular targets of GLP-1RAs

Our understanding of the disease mechanisms and molecular targets of drugs has been facilitated by new biotechnology advances, such as RNA sequencing. RNA-seq can also be used as a useful tool to dissect the molecular targets of drugs in CVD 176. Therefore, the use of new technological advances such as single cell RNA-seq can shed new light on the GLP-1RA-dependent and independent effects and targets in animal models and humans 177.

### The theranostic potential of new modifiers of GLP-1RAs

Due to the high expression profile of GLP-1R in pancreatic β-cells, GLP-1 analogs (such as exendin-4) have been modified by different methods (to prolong half-life, maintain receptor binding affinity and reduce renal drug accumulation) for theranostic purposes and serve as excellent templates for future cardiovascular drug development^178^-^184^. More importantly, these modified GLP-1 analogues have shown hypoglycemic 185, 186 and cardiovascular protective effects 180, 183 in different animal models. Further exploration of these modified agents in animal models of cardiometabolic diseases will deepen our understanding of the potential cardioprotective mechanisms of GLP-1 and GLP-1RAs and potentially provide future directions for precise therapeutic intervention 180.

GLP-1RAs are a class of agents with pleiotropic activities additional to the lowering of blood glucose. These pleiotropic effects include the reduction of BW and BP, the improvement of lipid metabolism and a spectrum of cardioprotective effects. The current comprehensive management strategy of diabetes has gradually shifted from “hypoglycemic as the focus” to “emphasizing both the control of blood glucose and the improvement of MACE and cardiovascular outcomes” 187, 188. In this regard, GLP-1RAs have shown promising efficacy as to the improvement of hard cardiovascular outcomes with cardiovascular safety.

## Conclusions

The ADA standard for the treatment of diabetes updated in 2020 indicated that patients with T2DM diagnosed with CVD or those with CVD risk factors can use GLP-1RAs and SGLT2 inhibitors with evidence of cardiovascular benefits to reduce cardiovascular risk, regardless of HbA1c levels or individualized HbA1c control objectives 189. This guideline emphasizes the importance of the primary and secondary prevention of CVD with both GLP-1RAs and SGLT2 inhibitors. It remains to be investigated whether cardiovascular protective effects of GLP-1RAs can also be independent of the known effects on glucose metabolism. In the future, more GLP-1RAs will be explored for the improvement of ischemic stroke and cognitive impairment in patients with diabetes 190. More importantly, endocrinologists should work coordinately with cardiologists and pharmacologists to establish GLP-1RA-dependent and independent effects in preparation for precisely commencing therapy with GLP-1RAs in suitable targeted patient categories.

## Figures and Tables

**Table 1:** Current GLP-1RAs in the clinic

GLP-1RAs	Classification	Frequency of administration	Half-life (t_1/2_)
Lixisenatide	Short-acting	Once daily	3 h
Oral semaglutide	Short-acting	Once daily	≈1 week
Exenatide	Short-acting Long-acting	Twice daily/ Once weekly	2.4 h/Sustained-release
Liraglutide	Long-acting	Once daily	13 h
Semaglutide	Long-acting	Once weekly	≈1 week
Albiglutide	Long-acting	Once weekly	≈5 days
Taspoglutide	Long-acting	Once weekly	≈1 week
Dulaglutide	Long-acting	Once weekly	4.5-4.7 days

**Table 2:** Cardiovascular outcome trials (CVOTs) of GLP-1RAs

GLP-1RAs	Trails	Patients	Median duration of follow-up	Outcomes			References
				MACE	CV Death	HHF	
Lixisenatide	ELIXA NCT01147250	6068 T2DM patients recently suffering acute coronary syndrome	2.1	1.02 (0.89-1.17)	0.98 (0.78-1.22)	0.96 (0.75-1.23)	47
Liraglutide	LEADER NCT01179048	9340 T2DM patients with CV risk factors or CVD	3.8	0.87 (0.78-0.97)	0.78 (0.66-0.93)	0.87 (0.73-1.05)	4
Semaglutide	SUSTAIN-6 NCT01720446	3297 T2DM patients with CV risk factors or CVD	2.1	0.74 (0.58-0.95)	0.98 (0.65-1.48)	1.11 (0.77-1.61)	22
Exenatide	EXSCEL NCT01144338	14752 T2DM patients with CV risk factors or CVD	3.2	0.91 (0.83-1.00)	0.88 (0.76-1.02)	0.94 (0.78-1.13)	26
Albiglutide	Harmony Outcomes NCT02465515	9463 T2DM patients with high CV risk.	1.6	0.78 (0.68-0.90)	0.93(0.73-1.19)	0.85 (0.70-1.04)	27
Dulaglutide	REWIND NCT01394952	9901 T2DM patients with CV risk factors or CVD	5.4	0.88 (0.79-0.99)	0.91 (0.78-1.06)	0.93 (0.77-1.12)	23
Oral semaglutide	PIONEER 6 Clinical Trials NCT02692716	3183 patients,most had cardiovascular or chronic kidney disease	1.3	0.79 (0.57-1.11)	0.49 (0.27-0.92)	0.86 (0.48-1.44)	52

**Table 3:** Cardiovascular effects and mechanisms of GLP-1RAs in rodents

Drugs	Animal model	Treatment dose and duration	Observations and mechanism	References
Lixisenatide	ApoE^-/-^Irs2 +/- mice with atherogenic diet	10 μg/kg/day via osmotic minipumps for one month	atheroma plaque size↓ M2 phenotype↓ inflammation↓ IL-6↓; STAT3↓ STAT1↓; NOS↓	91
Lixisenatide	Watanabe heritable hyperlipidemic (WHHL) rabbits	30 nmoL/kg/day via osmotic pumps for 3 month	plaque stabilization↑; plaque progression↓ modifying plaque composition inhibiting plaque growth	58
Liraglutide	ApoE^-/-^ and LDLr^ -/-^ mice with a western diet	1 mg/kg/day subcutaneously in ApoE^-/-^ mice for almost 3 months or LDLr^-/-^ mice for almost one month	atherosclerosis↓ Inflammatory Pathways	16
Liraglutide	Moderately uremic LDLr^-/-^ mice with a western diet	1000 μg/kg/day for 11 weeks via injections	atherosclerosis↓; kidney inflammation↓ infiltration of CD4+ and CD8+ T cells↓	60
Liraglutide	ApoE^-/-^ with a high-fat diet	400 μg/kg/day via osmotic pumps for one month	atherosclerosis↓; VSMCs proliferation↓ AMPK signaling↑; cells in G2/M↓	56
Liraglutide	ApoE^-/-^ mice with a fat-rich diet	300 μg/kg/daily for one month via injections	atherosclerosis↓ proinflammatory immune cell and mediators↓ marrow-derived macrophages↑	92
Liraglutide	T2DM rats with a high fat diet and small dosage streptozotocin injection	200 μg/kg/day for 3 months via percutaneous injections	diabetic atherosclerosis↓ microvesicles production, macrophage apoptosis, and ER stress↓	66
Liraglutide	Ldlr^-/-^ mice with a western diet	1 mg/kg/day subcutaneously for one month	endothelial dysfunction↓; inflammation↓ regulation of vascular remodelling	62
Liraglutide	ApoE^-/-^ mice	107 nmol/kg/day via osmotic pumps for one month	atherosclerosis↓; foam cell formation↓ ACAT1↓	93
Liraglutide	ApoE^-/-^ mice with a high-fat and -cholesterol diet	300 μg/kg/day via subcutaneous injections for 6 weeks	atherosclerotic lesion formation↓ MΦ1-like monocytes and macrophages↓ MΦ2↑	65
Liraglutide	T1DM rats with STZ	0.3 mg/kg/twice daily via subcutaneous injections for one month	oxidative stress, cardiac steatosis and apoptosis↓ AMPK-SIRT1↑	94
Liraglutide	Streptozotocin-induced hyperglycemic apolipoprotein ApoE^-/-^ mice	17 nmol/kg/day or 107 nmol/kg/day via subcutaneously implanted osmotic pumps for one month	lipid deposition, plaque volume and intraplaque macrophage accumulation↓ AMPK-independent↑	55
Liraglutide	ApoE^-/-^ mice	300 mg/kg/twice daily via s.c injections for one month	endothelial cell dysfunction↓ eNOS↑ ICAM-1↓	57
Liraglutide	ApoE^-/-^ mice with a fat-rich diet	300 µg/kg/twice daily via osmotic mini-pumps for one month	formation↓ progression of atherosclerotic plaque stability↑ GLP-1R-dependent manner	59
Liraglutide	ApoE^-/-^ male mice with a high-fat diet	0.4 mg/kg/day via subcutaneous injections for 9 weeks	atherogenesis↓ AGEs-induced RAGE↓	95
Exenatide	ApoE^-/-^ mice with a western diet containing 21.00% fat	300 µg/kg/twice daily for 3 months via subcutaneous injections	vascular aging and atherosclerotic plaque growth↓; APN↑ MMP-9 and MMP-2↓; TLR2, TLR4↓	96
Exenatide	Female APOE*3-Leiden.CETP mice with a western diet	50μg/kg/day for one month via an osmotic minipump	liver inflammation and atherosclerosis development↓ GLP-1 receptor-dependent	63
Exenatide	Rats with a high-fat diet containing 2% cholesterol	3 μg/kg/twice daily via subcutaneous injection for 3 and 6 months	contraction of AGE-induced ECs↑ endothelial barrier injury↓ RAGE/Rho/ROCK↓; GLP-1R/cAMP/ PKA↑	97
Exenatide	Rats with a high-fat diet (2% cholesterol) and streptozotocin	3 μg/kg/twice daily via subcutaneous injections for 3 months	endothelial function↑; aortic oxidative stress level↓ hoA/ROCK/NF-κB/IκBα↓; AMPK↑	98
Exenatide	C57BL/6 or ApoE^-/-^ mice	low-dose (300 pmol/kg/day) and high-dose (24 nmol/kg/day) for one month via a mini-osmotic pump	monocytic adhesion↓ TNF α, monocyte chemoattractant protein-1, p65, and NF-κB↓	64
Semaglutide	ApoE^-/-^ and LDLr^ -/-^ Mice with a western diet	4.0, 12.0, or 60.0 mg/kg/day in ApoE^-/-^ mice for almost 3 months or in LDLr^-/-^ mice for almost 4 months	atherosclerosis↓ inflammatory pathways	16

## Abbreviations

ABCA1: ATP-binding cassette transporter A1
ACAT: acyl-CoA cholesterol acyltransferase
AMPK: AMP-activated protein kinase
APN: adiponectin
ApoE^-/-^: apolipoprotein E-deficient
APPL: activating the leucine zipper motif
ASC: apoptotic speck containing protein
Bax: Bcl-2-associated x
Bcl: B-cell lymphoma
BP: body pressure
BW: body weight
CaMK: calmodulin-dependent protein kinase
cAMP: cyclic adenosine monophosphate
CD31: cell adhesion molecule
C/EBP β: CCAAT/enhancer-binding protein β
CREB: cAMP response element binding-protein
CKD: chronic kidney disease
CRP: C-reactive protein
CTL: cytotoxic T lymphocyte
CVD: cardiovascular diseases
CVOT: cardiovascular outcomes trials
CXCR: C-X-C motif receptor
eNOS: endothelial NO synthase
ERK: extracellular signal-regulated kinase
FOXO: forkhead box O
GLP-1RA: receptor agonists for hormone glucagon like peptide-1
HDAC4: histone deacetylase 4
HO-1: heme oxygenase-1
ICAM-1: intracellular adhesion molecule-1
IFN: interferon
IL: interleukin
iNKT: invariant natural killer T
iNOS: inducible NOS
JAK: Janus kinase
JNL: Jun NH2-terminal kinase
KLF2: Kruppel-like factor 2
LDLr^-/-^: low-density lipoprotein receptor-deficient
MACE: major cardiovascular events
MAPK: mitogen-activated protein kinases
MGL-1: macrophage galectin-1
MI: myocardial infarction
MMP: matrix metalloproteinase
MRC-1: mannose receptor-1
mTOR: mechanistic target of rapamycin
NF-κB: nuclear factor-κB
NLRP3: Nod-like receptor protein 3
NOR1: neuron-derived orphan receptor 1
Nox4: NADPH oxidase 4
Nrf2: nuclear factor erythroid 2-related factor 2
ox-LDL: oxidized-LDL
PAI: plasminogen activator inhibitor
PARP-1: poly(ADP-ribose) polymerase 1
PINK1: the mitochondrial kinase
PI3K: phosphoinositide 3-kinase
PKA: protein kinase A
PPAR: peroxisome proliferator-activated receptor
P10: cleaved caspase 1
P4HA1: prolyl 4-hydroxylase subunit alpha-1
p70S6K: p70 ribosomal protein S6 kinase
Rac1: Ras-related C3 botulinum toxin substrate 1
RAGE: receptor AGE
RANKL: receptor activator of nuclear factor kappa B ligand
Rho: the small GTPase
ROCK: Rho kinase
ROS: reactive oxygen species
RyR2: the type 2 ryanodine receptor
SDF: stromal cell-derived factor
SREBP1: element binding transcription factor 1
SIRT: sirtulin
α-SMA: alpha smooth muscle actin
SM22α: sensitive 22 kDa actin-binding protein of the calponin
STAT: cAMP-PKA-signal transducers and activators of transcription
TGF: transforming growth factor
TIMP: tissue inhibitor of MMPs
TNF: tumor necrosis factor
Treg: regulatory T cell
TXNIP: thioredoxin-interacting protein
T2DM: type 2 diabetes mellitus
VAM: vascular adhesion molecule
VCAM-1: vascular cell adhesion molecule 1
VSMC: vascular smooth muscle cells
